# Nano-selenium mitigates arsenate toxicity in soybean roots by modulating phenylalanine and salicylic acid pathways

**DOI:** 10.1186/s12870-025-06726-0

**Published:** 2025-05-26

**Authors:** Muhammad Zeeshan, Aamir Hamid Khan, Abdul Salam, Yuxin Hu, Anas Iqbal, Ruiquan Hou, Abdul Wakeel Umar, Feibo Wu, Xiaoyuan Chen, Zhixiang Zhang

**Affiliations:** 1 State Key Laboratory of Green Pesticide, South China Agricultural University, Guangzhou, China; 2 Yingdong College of Biology and Agriculture, Shaoguan University, Shaoguan, China; 3 Department of Biogeography, Paleoecology and Nature Conservation, Faculty of Biology and Environmental Protection, University of Łódź, Banacha 1/3, Łódź, 90-237 Poland; 4 College of Pastoral Agriculture Science and Technology, Lanzhou University, Lanzhou, China; 5 Guangzhou Key Laboratory for Science and Technology of Fragrant Rice, Guangzhou, 510642 China; 6 BNU-HKUST Laboratory of Green Innovation, Advanced Institute of Natural Sciences, Beijing Normal University at Zhuhai (BNUZ), Zhuhai City, 519087 People’s Republic of China; 7 Department of Agronomy, College of Agriculture and Biotechnology, Zhejiang University, Zijingang Campus, Hangzhou, 310058 China

**Keywords:** *Glycine max*, Selenium nanoparticles, Arsenic stress, Hormonal regulation, Transcriptomic

## Abstract

**Background:**

Soybean (*Glycine max* L. Merrill), a vital source of edible oil and protein, ranks seventh in global agricultural production, yet its productivity is significantly hindered by potential toxic metal/liods (PTM) stress. Arsenic (As), a highly toxic soil contaminant, poses substantial risks to both plants and humans, even at trace concentrations, particularly in China.

**Results:**

This research endeavor delves into the combined effect of arsenate (AsV), a common form of As in soil, and nano-selenium (nSe), on the transcriptional regulation of key genes and the modulation of signaling and metabolic cascades in young soybean seedlings. Our findings indicate that nSe mitigates AsV toxicity by modulating hormonal signaling cascades, particularly the phenylalanine and salicylic acid pathways, thereby augmenting antioxidant defenses and mitigating the damaging effects of reactive oxygen species (ROS) on soybean roots.

**Conclusion:**

This study offers valuable insights into the molecular mechanisms underlying metalloid tolerance in soybean, opening avenues for the development of strategies to bolster As resistance in contaminated soils. Nevertheless, further investigation is imperative to elucidate the intricate interplay of hormonal signaling in soybean roots during nSe supplementation under As stress conditions.

**Supplementary Information:**

The online version contains supplementary material available at 10.1186/s12870-025-06726-0.

## Background

Engineered nanoparticles (ENPs) and heavy metal/loids also called potential toxic metal/loid (PTM) represent two significant environmental concerns that have garnered considerable attention and scrutiny within the scientist community over the past decade. The rapid expansion of the nanotechnology industry, with forecasts predicting its growth to reach $3 trillion in final goods in coming decades [1], has resulted in a substantial increase in the synthesis of ENPs. This, in turn, has led to a rise in their disposal and unintended releases into the environment [2]. Concurrently, both anthropogenic and natural phenomenon have contributed to heightened concentrations of PTM accumulation in the soil, which pose detrimental effects to both flora and fauna resulting in environmental contamination and accumulation in the food chain [3].

Among the many nanoparticles (NPs) available today, elemental selenium nanoparticles (SeNPs) represent a promising avenue for reducing environmental harm while simultaneously enhancing crop yield, under (a)biotic stress [4, 5]. Selenium nanoparticles exhibit reduced cellular toxicity and possess distinctive physicochemical properties and bioactivities, thus demonstrating a high level of biosafety [6, 7]. Furthermore, the nanoscale dimensions and spherical morphology of SeNPs, which mimic biomolecular characteristics, facilitate their penetration through cell membranes and enable their intracellular functions [7, 8]. These nano-sized Se particles, formed through the reduction of Se oxyanions via biological or non-biological processes, are prevalent in the environment, particularly in regions with higher heavy metal mining activities [9].

Similarly, arsenate (As) is one of the most toxic and threatening PTM in soil particularly in China [10]. Its toxicity extends across all life forms, including humans and plants, and even minute concentrations can lead to various carcinogenic and non-carcinogenic risks. The issue of As pollution has escalated into a significant global environmental concern, with over 20 countries and regions, predominantly in South and Southeast Asia, experiencing high levels of contamination [3, 11, 12]. The presence of this semi-metal in irrigation water, which is applied for agricultural purposes, has raised considerable alarm within the global community. This concern stems from the documented absorption of As by plants, leading to phytotoxicity and the contamination of the food chain [13].

Phenylalanine is an aromatic amino acid that serves as the carbon backbone for the phenylpropanoid pathway. This pathway produces a wide variety of compounds involved in defense mechanism, structural integrity, and potentially other undiscovered functions [14]. Salicylic acid (SA), a plant growth regulator, plays a diverse role in plants including influencing biochemical processes, development, and photosynthetic mechanism [15]. Except these functions, SA also mediates plant responses to various stresses such as arsenic stress [16]. Various studies have shown that exogenous application of SA enhanced growth and photosynthesis in several crops such as *Artemisia annua* [17], *Oryza sativa* [18], and *Glycine max* [19].

Recent evidences have demonstrated that PTM and ENPs interact synergistically, influencing their transport and fate within soil-plant systems [20].For example, the combined application of SeNPs, zinc oxide nanoparticles (ZnONPs), and As stress has been shown to modify the physiological and biochemical characteristics of soybean plants and affect the As uptake into plant tissue [21, 22]. The addition of SeNPs to cadmium (Cd)-stressed *Brassica napus* plants has been found to alleviate Cd toxicity and enhance plant resilience [23]. Likewise, seed priming with ZnONPs has been observed to decrease the uptake and bioaccumulation of cobalt (Co) in maize crops, promote the detoxification of reactive oxygen species and lipid peroxidation caused by Co stress, resulting in improved chloroplast integrity, enhanced nutrient acquisition, and protection of cellular structures [24]. Furthermore, positive effects of titanium dioxide nanoparticles (TiO_2_) on the photosynthetic machinery, antioxidative systems, and modulation of stress-responsive gene expression in *Oryza sativa* L. under Cd toxicity have also been reported [25]. In addition, our previous study identified that, ZnONPs supplementation into the AsV-stressed plant augmented the expression of genes responsible for several signaling pathways including jasmonic acid, gibberellin, abscisic acid, and auxin [26]. Nonetheless, the mechanistic understanding of pathways involved in the accumulation of PTM and plant molecular responses and signaling transduction in the presence of NPs are yet remains unclear and requires further investigation.

Soybean, a significant source of edible oil and protein, ranks as the seventh most produced crop globally, as reported by the Food and Agriculture Organization of the United Nations (FAOSAT, www.fao.org). Unfortunately, this crop is vulnerable and exposed to both PTM [27], as well as ENPs [28]. Consequently, it is imperative to investigate the interplay between PTM and ENPs in soybean. The primary goals of this research were: (i) to ascertain the combined impact of AsV and nSe on the expression of potential target genes and the modulation of signaling and metabolic pathways in young soybean seedlings, and (ii) to elucidate the molecular mechanisms underlying the soybean plant’s ability to coordinate multi-gene complexes to mitigate AsV toxicity in the presence of nSe. The authors posit that this study represents the first RNA-seq investigation to employ nSe for the alleviation of AsV-induced toxicity in soybean roots.

## Materials and methods

### Plant material and stress treatment

The soybean genotype “ZhongHoung302”, utilized in this study, was obtained from the Guangxi Agricultural Research Center, located in Nanning, within the Guangxi Zhuang Autonomous Region. Seeds were grown in sterile vermiculite for ten days under temperature conditions of 25 °C to 28 °C and a light intensity of 250 to 300 µmol m^− 2^ s^− 1^, provided by artificial lighting with a 15/9-hour day/night photoperiod. The seedlings were then rinsed and transferred to 10 L pots containing half-strength Hoagland solution consisting of 0.6 mM KNO_3_, 0.28 mM MgSO_4_, 0.13 mM KH_2_PO_4_, 0.084 mM KCl, 0.24 mM Ca(NO_3_)_2_, 2.3 µM ZnSO_4_, 4.5 µM KI, 0.003 µM Na_2_MoO_4_, 28 µM MnCl_2_, 19 µM H_3_BO_3_, 0.5 µM CuSO_4_, and 2 µM Fe-EDTA, and pH was set at 6.0 as suggested by Sugiyama et al. [29]. Soybean plants were grown for 5 days in Hoagland solution until the first two unfolded trifoliolate leaf nodes developed, indicating the V2 growth stage. At the V2 growth stage, four different treatments were applied: Control (CK: only nutrient supply), AsV (Na_2_HAsO_4_ stress: 25 µmol L^− 1^), nSeA + AsV (SeNPs 10 µmol L^− 1^ + AsV), and nSeB + AsV (SeNPs 25 µmol L^− 1^ + AsV). The Na_2_HAsO_4_ and nSe were purchased from Sigma Aldrich, USA, and used as received. After 10 days of treatment, roots were collected in liquid nitrogen and stored at -80 °C for further experiments. The treatments concentration was selected based on our previous study. More detail of the materials and methods can be found in our previous study [22].

### Endogenous hormone quantification and analysis in soybean root tissues

The quantification of endogenous phytohormones, including gibberellic acid (GA_3_), abscisic acid (ABA), indole acetic acid (IAA), salicylic acid (SA), methyl jasmonate (MeJA), and zeatin riboside (ZR), in soybean root under different treatment condition were quantified using a protocol proposed previously [30].For instance, root sample were homogenized by tissue homogenizer in liquid nitrogen and 50 mg crushed sample were transferred to 2 ml tube. The internal standards of the above phytohormones such as d_2_-GA_3_, d_6_-ABA, d_5_-IAA, d_5_-SA, MeH_2_ JA and d_5_-ZR (Shanghai, Yuanye Bio-Technology Co., Ltd., Shanghai, China) were added into the root samples along with 0.5 mL of extraction solution consisting of water/2-propanol/HCl (1:2:0.002 vol/vol/vol). Each tube containing mixture was putted on shaker at 100 rmp at 4 °C for 30 min. After adding 1 ml dichloromethane to each tube, the tubes were shaken again for 30 min at 4 °C. Afterward, the samples were centrifuged at a speed of 13,000 g for 30 min at 4 °C. About 900 ul of supernatant were collected and dried with nitrogen evaporator and again redissolved in 100 µl of methanol. After a brief centrifugation, the samples were ready for LC-MS analysis. Detail about LC-MS setup and operating procedure can be seen in Pan et al. [30].

### RNA extraction and sequencing

To further determined the effect of stress on soybean roots, root samples were collected from both CK and treated groups. RNA was extracted (1.5 g per sample) from eight samples (including biological replicates) categorized as CK, AsV, nSe 10 µmol L^− 1^+AsV and nSe 25 µmol L^− 1^ + AsV using TRIzol^®^ following the Zeeshan et al., (5) method. RNA was then purified from DNA contamination using RNase-free DNase 1. Bioanalyser and agarose gel electrophoresis was used to check the RNA quality, while spectrophotometer was used to assessed the concentration of RNA. Samples were sent to the Majorbio sequencing platform for library preparation and illumina sequencing.

The mRNA was supplemented by using oligo (dT) beads and divide into fragments for the synthesis of cDNA by using the SuperScript double stranded cDNA synthesis kit (Invitrogen, CA). The cDNA was polyadenylated, the sequences were randomly fragmented (170–300 bp), and then amplified using Phusion DNA polymerase before sequencing on the Illumina NovaSeq 6000 sequencer.

### Data analysis

Clean data were obtained after removing connector sequences, low-quality bases, and unknown nucleotides. The clean data were then mapped to the Glycine max reference genome (version Wm82.a4; available at https://data.jgi.doe.gov/refine-download/phytozome? genome_id = 508) using HISAT2 software (http://ccb.jhu.edu/software/hisat2/index.shtml). In each library, the mapped data were analyzed using StringTie software. Gene expression levels were measured using RSEM software (http://deweylab.github.io/RSEM/) and differentially expressed genes (DEGs) were identified through DESeq2 (http://bioconductor.org/packages/stats/bioc/DESeq2/) by adjusting screening criteria (q-values < 0.001 and |log2FC| ≧ 1). For Gene ontology (GO) and Kyoto Encyclopedia of Genes and Genomes (KEGG) analysis, Diamond (GO, https://github.com/bbuchfink/diamond) and ID mapping (KEGG) were used, respectively.

### Quantitative reverse transcription-PCR

To assess the RNA-seq data, 11 genes are randomly selected from DEGs and quantitative reverse transcription-PCR (qRT-PCR) was conducted using the SYBR Green Mastermix protocol (Applied Biosystems, Waltham, MA, USA). Genes specific primers were design using primer premier 5.0 (Primer, Palo Alto, CA, USA) (Supplementary Table S1). Two independent biological and two technical replicates were used. The same RNA templates used in library construction were also utilized for this experiment, while cDNA synthesis was performed using the SuperMix First-Strand Synthesis kit (TransGen Biotech Co., Ltd, Beijing, China). The qRT-PCR step involved were 1 cycle of 95 °C for 5 min for initial denaturation, followed by 45 cycles for 10 s at 95 °C for amplification and 20 s at 55 °C and 72 °C each, melting for 1 min at 95 °C and 65 °C each and 95 °C continuous; and cooling for 30 s at 40 °C. Relative expression of the obtained CT values were computed by a formula 2^−ΔΔCT^ suggested by Livak and Schmittgen [31]. In order to normalize the data, *Gm*Tubulin (NM_001358288.1) was used as a reference gene.

### Statistical analysis

Phytohormones data were presented as mean ± SD from three independent biological replications per treatment. One-way ANOVA (analysis of variance) was used for statistical analysis, and different small letters above the bars indicate significant differences (*p* < 0.05) using the Tukey HSD test. Graphs were created using OriginPro 2022 software.

## Results

### Transcriptomic analysis and identification of DEGs in soybean roots

**Fig. 1:**
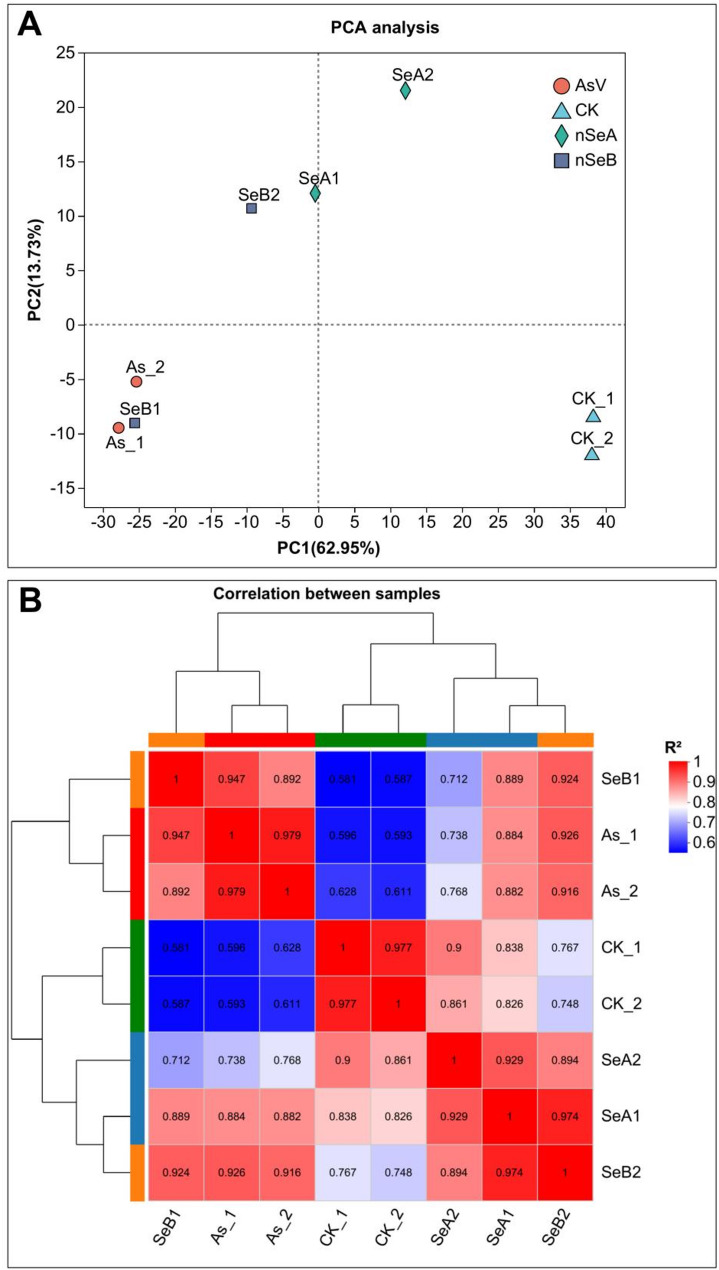
Principal component and correlation coefficient analysis of AsV and nSe treatment samples. **(A)** Represent the Principal component analysis develop on the values of TPM. **(B)** Represent the correlation coefficient analysis. Various colors shown the correlation analysis between samples. The scale bar represents the low and high correlation between samples. CK (control), As = AsV (arsenate stress), nSeA = SeA (10 µmol L^− 1^ SeNPs + AsV), nSeB = SeB (25 µmol L^− 1^ SeNPs + AsV), SeNPs (Selenium nanoparticles)

**Fig. 2:**
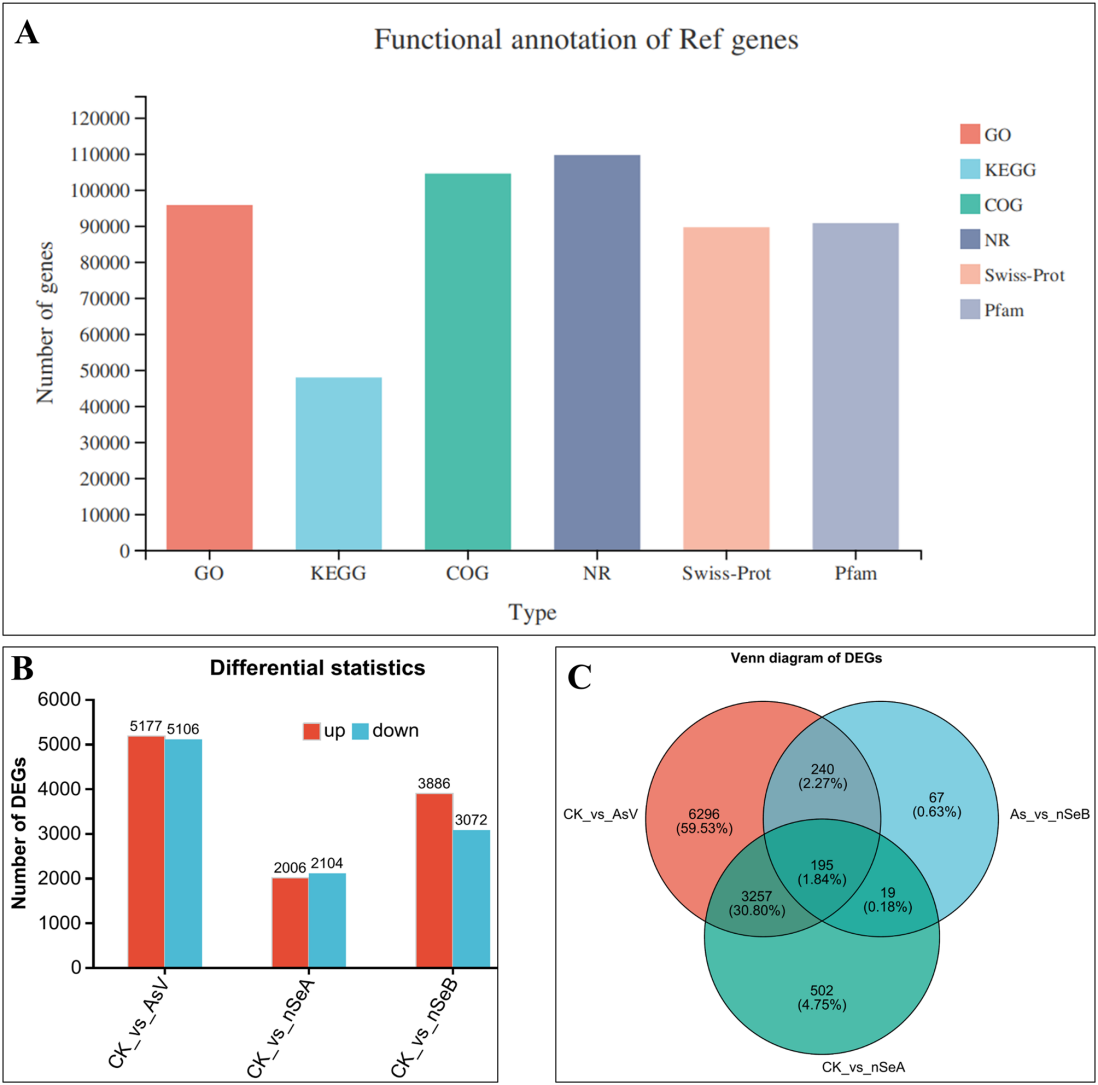
Functional annotation, differential statistics and venn diagram of DEGs in soybean roots after treatment with AsV and nSe. **(A)** Bar plots represent the functional annotation of all genes. **(B)** Bar plots represent the significantly up and down-regulated genes in all treatments. **(C)** Venn diagram represent the unique and common DEGs in all treatments. The various treatment groups include CK (control), AsV (arsenate stress), nSeA (10 µmol L^− 1^ SeNPs + AsV), nSeB (25 µmol L^− 1^ SeNPs + AsV), SeNPs (Selenium nanoparticles)

RNA-seq was performed on soybean roots under four different treatments: Control (no nutrient supply), arsenate stress (AsV), nSeA (nSe 10 µmol L^− 1^+AsV) and nSeB (nSe 25 µmol L^− 1^+AsV). Clean reads were generated from the raw reads of 8 samples (4 treatments with 2 biological replicates each), yielding a total read range of 48,259,198 to 63,900,740. By mapping the reads against the soybean reference genome, we identified a total of 41,782,386 to 56,800,033 mapped sequences, with 1,164,118 to 1,728,284 sequences mapped to multiple locations, and 40,618,268 to 55,258,059 uniquely mapped sequences (Table 1). The GC content (%) for all samples libraries ranged from 45.42 to 45.82% while the base quality scores, Q20 (Phred > 20) and Q30 (Phred > 30), ranged from 97.62 to 97.92% and 93.12–93.91%, respectively. In the principal component analysis (PCA) of all treatments, the reproducibility indices showed a high segregation of 62.95% in PC1 (principal component 1), as well as noticeable segregation in PC2 (principal component 2) among all treatments (Fig. 1A). A pearson correlation coefficient analysis demonstrated high validity between repetitions (R^2^ > 0.979) (Fig. 1B). In functional annotation, the genes showed strong alignment to the NR (98.28%) and COG (91.95%) databases (Fig. 2A).

**Table 1:** Table represent the summary of the read numbers of transcriptomic (RNA-seq) data

Sample	Total reads	Total mapped	Multiple mapped	Unique mapped	Q20%	Q30%	GC%
AsV_1	49,648,720	44,006,087(88.63%)	1,290,490(2.6%)	42,715,597(86.04%)	97.92	93.91	45.55
AsV_2	53,017,064	47,509,322(89.61%)	1,540,667(2.91%)	45,968,655(86.71%)	97.62	93.21	45.56
CK_1	57,952,486	53,764,148(92.77%)	1,561,163(2.69%)	52,202,985(90.08%)	97.92	93.76	45.46
CK_2	51,121,426	47,345,705(92.61%)	1,309,869(2.56%)	46,035,836(90.05%)	97.58	93.12	45.42
nSeA1	48,259,198	41,782,386(86.58%)	1,164,118(2.41%)	40,618,268(84.17%)	97.86	93.71	45.51
nSeA2	61,543,042	55,736,427(90.56%)	1,728,284(2.81%)	54,008,143(87.76%)	97.75	93.5	45.82
nSeB1	63,900,740	56,800,033(88.89%)	1,541,974(2.41%)	55,258,059(86.47%)	97.82	93.69	45.43
nSeB2	57,828,250	53,050,056(91.74%)	1,517,146(2.62%)	51,532,910(89.11%)	97.81	93.56	45.59

**Fig. 3:**
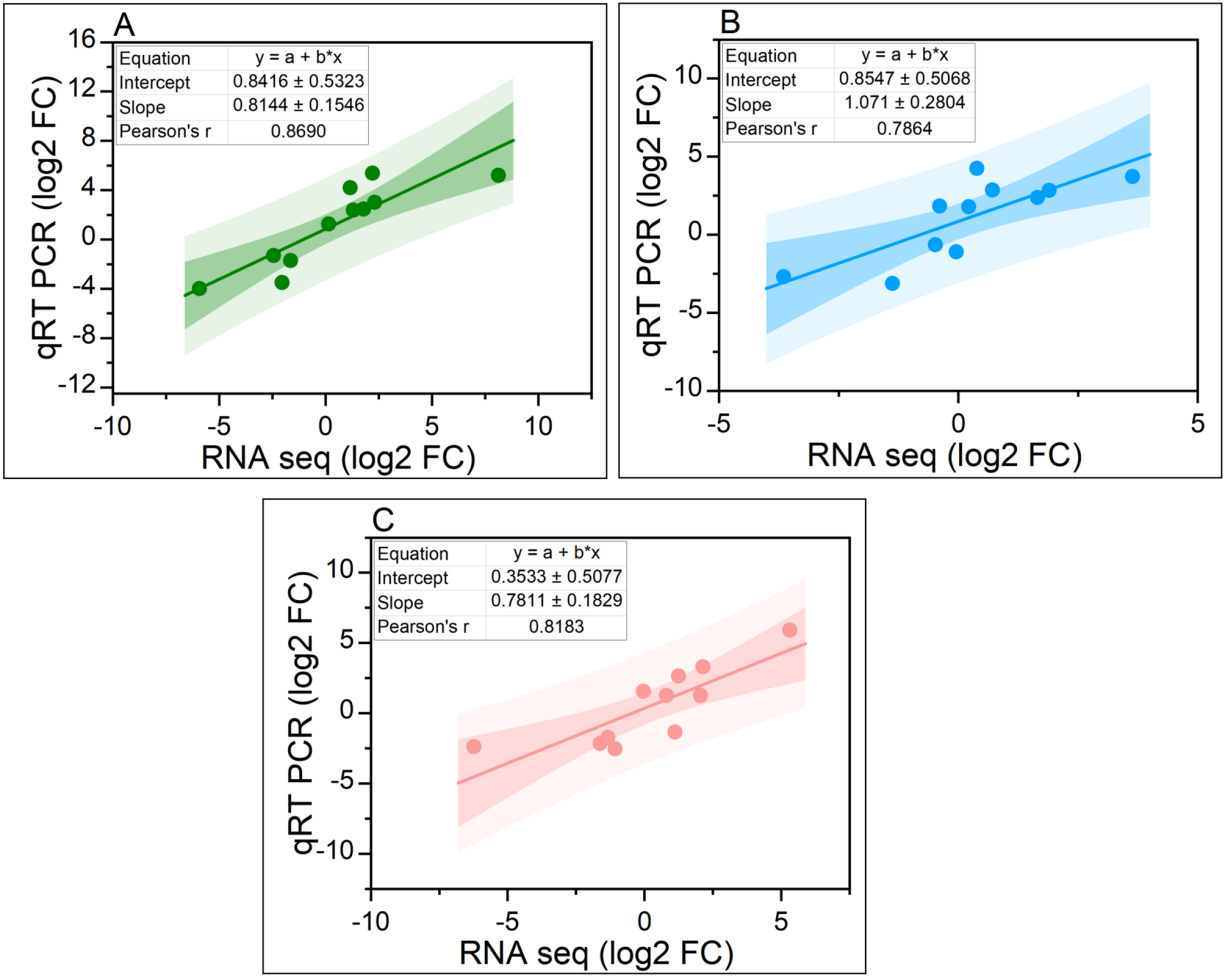
Regression correlation between expression level changes observed in 11 randomly selected DEGs (**A**) Ck_vs_AsV, (**B**) Ck_vs_nSeA + AsV and (**C**) Ck_vs_nSeB + AsV analyzed by RNA-seq (X-axis) and qRT-PCR (Y-axis)

To study gene expression levels, read count and gene length were used to calculate the Transcripts Per Million (TPM) values. Based on TPM values, a total of 21,351 DEGs were identified across all the samples, with TPM ≥ 0 (q-values < 0.001) and an absolute value of log2FC ≧ 1 with a false discovery rate < 0.001. In total, 21,351 DEGs were identified, with a higher number of DEGs found in CK vs. AsV (5,177 up-regulated and 5,106 down-regulated), compared to CK vs. nSeA (2,006 up-regulated and 2,104 down-regulated). The number of DEGs found in CK vs. nSeB (3,886 up-regulated and 3,072 down-regulated) fell between the numbers observed in CK vs. AsV and CK vs. nSeA (Fig. 2B). In the venn diagram, 3,668 DEGs were uniquely found in CK vs. AsV, 352 were unique to CK vs. nSeA and 520 DEGs were unique to CK vs. nSeB. A high number of common DEGs (2868) were identified between CK vs. AsV and CK vs. nSeB, while lower number of common DEGs (169) were found between CK vs. nSeA and CK vs. nSeB (Fig. 2C). In addition, in our study, regression analysis was employed to examine the relationship between the qRT-PCR and RNA-Seq data results. The results revealed a significant positive linear correlation for AsV (R2 = 0.8690), nSeA (R2 = 0.7864), and ZnOB (R2 = 0.8183), as illustrated in Figs. 3A-C. This robust correlation provides strong evidence to support the reliability of the RNA-seq data obtained in this investigation.

### GO annotation and KEGG enrichment analysis

**Fig. 4:**
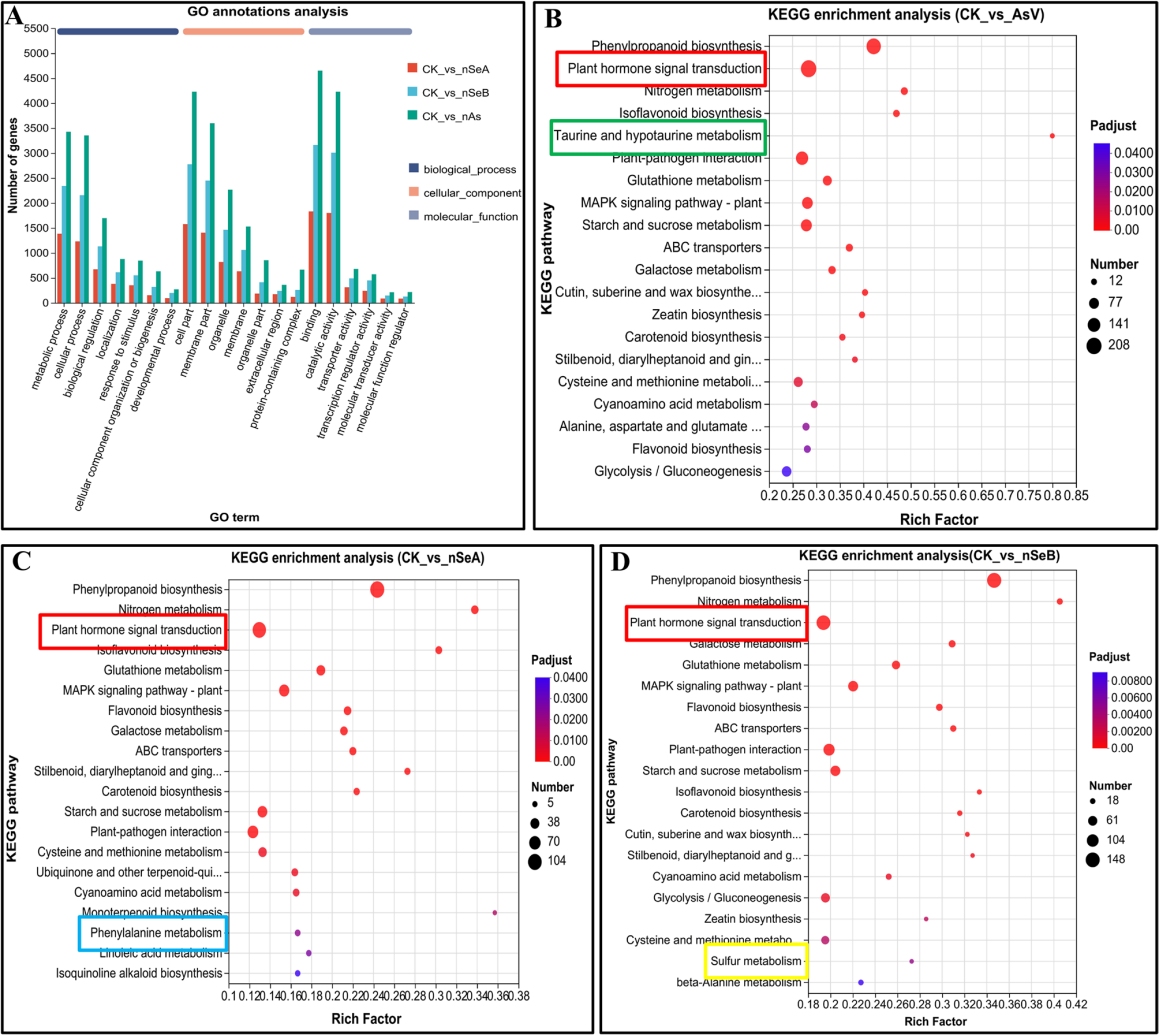
GO annotation and KEGG enrichment analysis of AsV and nSe treatment samples. **(A)** Represent the GO annotation analysis of CK vs. AsV, CK vs. nSeA and CK vs. nSeB treatments. **(B)** Represent the KEGG enrichment analysis of CK vs. AsV. **(C)** Represent the KEGG enrichment analysis of CK vs. nSeA. **(D)** Represent the KEGG enrichment analysis of CK vs. nSeB. The various treatment groups include CK (control), AsV (arsenate stress), nSeA (10 µmol L^− 1^ SeNPs + AsV), nSeB (25 µmol L^− 1^ SeNPs + AsV

To further investigate the potential roles of DEGs, a GO enrichment analysis was conducted for all samples. The DEGs were categorize into 3 groups: biological process, cellular component and molecular function. Among these categories, CK vs. AsV exhibited the highest number of DEGs, whereas CK vs. nSeA showed the lowest number of DEGs. Furthermore, metabolic process, cell part, and binding activity were prominent in the biological process, cellular component, and molecular function categories, respectively (Fig. 4A). In a subsequent study, DEGs were analyzed using ID mapping (KEGG) software to develop KEGG enrichment plots for all samples. In the KEGG enrichment plot, various pathways were significantly enriched and were further selected for the identification of significantly affected genes under the stress conditions. Among these pathways, phenylpropanoid biosynthesis and plant hormone signal transduction were highly significantly enriched across all treatment conditions. Conversely, taurine and hypotaurine metabolism, phenylalanine metabolism, and sulfur metabolism were only enriched in the CK vs. AsV, CK vs. nSeA, and CK vs. nSeB treatments, respectively (Fig. 4B-D).

### nSe maintains the plant hormone signal transduction pathway in soybean roots under AsV toxicity

**Fig. 5:**
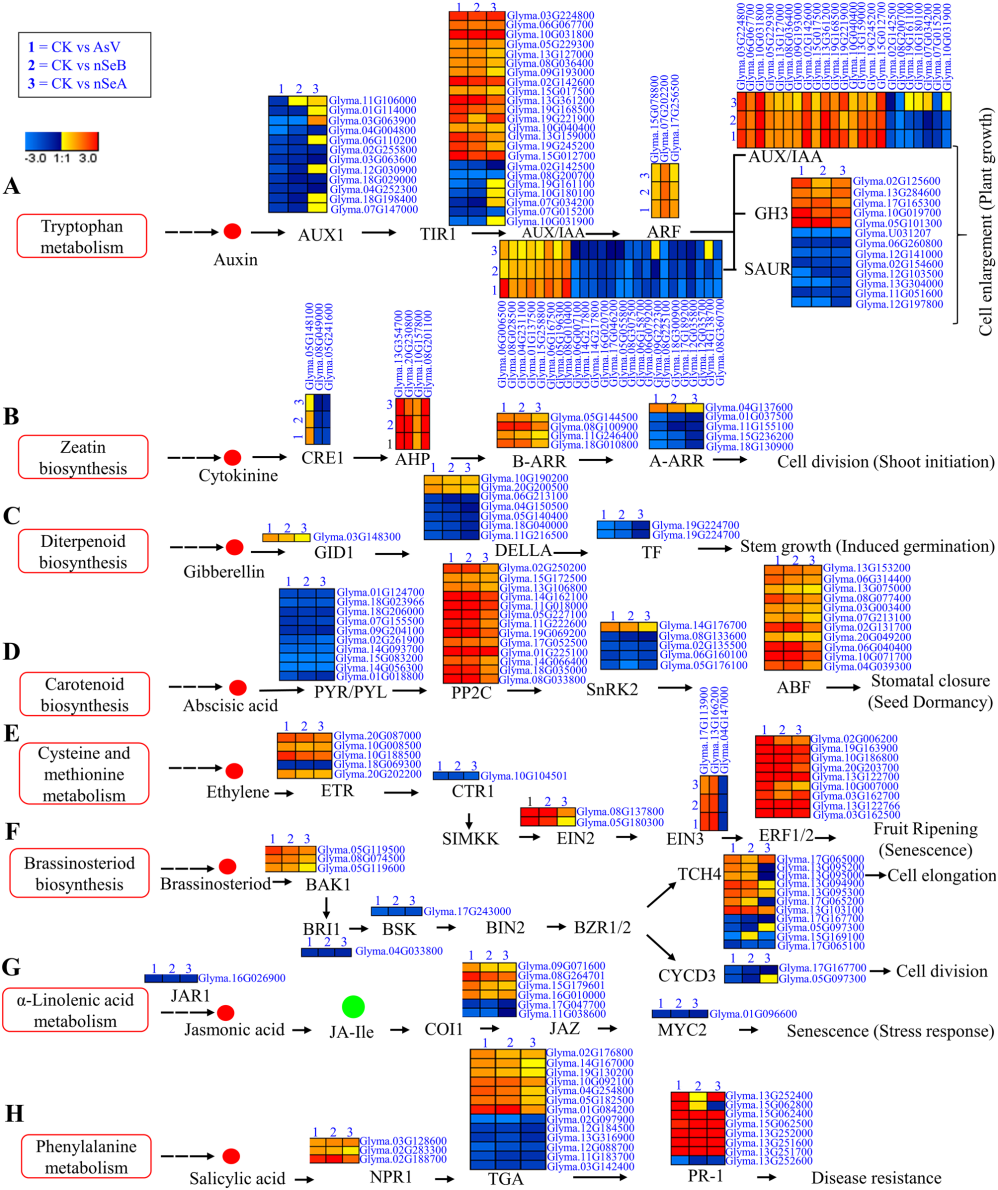
Analysis of hormonal signaling pathways and corresponding transcriptional responses in soybean roots under AsV stress and nSe supplementation at varying concentration. **(A)** Auxin signaling pathway and differential expression of related transcripts in response to AsV stress and nSe supplementation. **(B)** Cytokinin signaling pathway and its transcriptional modulation under AsV stress conditions, with and without the addition of nSe. **(C)** Gibberellin signaling cascade and its transcriptome dynamics during AsV-induced stress, in comparison to treatments involving nSe supplementation. **(D)** Abscisic acid signaling pathway and the differential expression patterns of associated transcripts in response to AsV Stress and nSe supplementation. **(E)** Ethylene signaling pathway and the differential expression patterns of associated transcripts in response to AsV Stress and nSe supplementation. **(F)** Brassinosteroid signaling pathway and its transcriptional reprogramming in soybean roots in response to AsV stress and nSe supplementation. **(G)** Jasmonic acid signaling cascade and the differential expression of related genes during AsV Stress and nSe supplementation. **(H)** Salicylic acid signaling pathway and its transcriptional alteration in soybean roots exposed to AsV stress, with and without nSe supplementation. The heatmaps presented depict the log2 fold change values of differentially expressed genes (DEGs), facilitating the visualization of transcriptional alterations across various treatment groups: CK (untreated control), AsV (arsenate stress alone), nSeA (10 µmol L^− 1^ SeNPs plus AsV), and nSeB (25 µmol L^− 1^ SeNPs plus AsV). *Jasmonate-zim domain* (*JAZ*), *Myelocytomatosis-2* (*MYC2*), A*uxin Resistant 1* (*AUX1*), *Auxin/Indole-3-Acetic Acid* (*AUX/IAA*), small auxin up-regulated RNAs (SAUR), *Cytokinin Response1* (*CRE1*), *Gibberellin Insensitive Dwarf1* (*GID1*), *Sucrose Non-Fermenting 1* (*SNF1*)-related Protein Kinase 2s (SnRK2s), *Ethylene-Insensitive3* (*EIN3*), BRI1-associated receptor kinase1 (BAK1), *cyclinD3* (*CYCD3*), *xyloglucan endotransglucosylase* (*TCH4*), *nonexpressor of PR1* (NPR1), TGACG motif-binding proteins (TGA), *pathogenesis-related class 1* (*PR-1*), Auxin response factor (ARF), Transport inhibition response 1 (TIR1), Gretchen hagen 3 (GH3), Arabidopsis histidine phosphotransfer proteins (AHP)

Plant hormones play a vital role under stress condition [32, 33]. In our previous research, we found that As stress affects the plant hormone pathway genes in soybean roots. Our current study also revealed through transcriptional profiling that various DEGs were significantly altered in the plant hormone signal transduction pathway across all treatment conditions (Supplementary Table 2). In the auxin signaling pathway, the *auxin resistant 1* (*AUX1*) gene (*Glyma.11G106000.Wm82.a4.v1*) was differentially regulated in the CK vs. AsV, CK vs. nSeA, and CK vs. nSeB treatments. Additionally, 6 *AUX1* genes were down-regulated in the CK vs. AsV and CK vs. nSeB treatments, but their expression were normal in the CK vs. nSeA treatment, except for *Glyma.03G063900.Wm82.a4.v1* which was upregulated (Fig. 5A). Likewise, four *Auxin/Indole-3-Acetic Acid* (*AUX/IAA*) genes exhibited unchanged expression after the supplementation of nSeA, compared to AsV stress and nSeB supplementation. All these genes (*Glyma.19G161100.Wm82.a4.v1*, *Glyma.10G180100.Wm82.a4.v1*, *Glyma.07G034200.Wm82.a4.v1* and *Glyma.10G031900.Wm82.a4.v1*) were down-regulated under AsV stress and nSeB treatment. Additionally, genes in the auxin family, such as small auxin up-regulated RNAs (SAUR), also showed down-regulation during AsV stress. Among these down-regulated genes, only two (*Glyma.14G138700.Wm82.a4.v1* and *Glyma.09G222300.Wm82.a4.v1*) exhibited unchanged expression after supplementation with nSeA. Moreover, five *SAUR* genes were up-regulated during AsV stress. Of these, four (*Glyma.08G028500.Wm82.a4.v1*, *Glyma.04G231100.Wm82.a4.v1*, *Glyma.15G258800.Wm82.a4.v1* and *Glyma.05G196300.Wm82.a4.v1*) showed unchanged expression after supplementation with nSeA, while one (*Glyma.06G006500.Wm82.a4.v1*) showed unchanged expression after supplementation with nSeB. In the cytokinine, gibberellin, and abscisic acid signaling pathways, only one gene from each category showed variation. *Cytokinin Response1* (*CRE1*), *Gibberellin Insensitive Dwarf1* (*GID1*) and *Sucrose Non-Fermenting 1* (*SNF1*)-related Protein Kinase 2s (SnRK2s) genes were up-regulated during AsV stress, but their expression remained unchanged after supplementation with nSeA (Fig. 5B-D). In ethylene signaling pathway, only one *Ethylene-Insensitive3* (*EIN3*) (*Glyma.05G180300.Wm82.a4.v1*) and one *ERF1/2* gene (*Glyma.10G007000.Wm82.a4.v1*) were up-regulated during AsV stress, while both genes showed unchanged expression after supplementation with nSeA (Fig. 5E).

In the brassinosteriod signaling pathways, only one BRI1-associated receptor kinase1 (BAK1) (*Glyma.05G119600.Wm82.a4.v1*) and one *cyclinD3* (*CYCD3*) gene (*Glyma.05G097300.Wm82.a4.v1*) were up-regulated during AsV stress, while both genes showed unchanged expression after supplementation with nSeA. Four genes in the brassinosteriod family such as *xyloglucan endotransglucosylase* (*TCH4*), showed down-regulation, while seven genes were up-regulated during AsV stress. Among the down-regulated genes, only one *TCH4* gene (*Glyma.05G097300.Wm82.a4.v1*) showed unchanged expression after supplementation with nSeA, and one *TCH4* gene (*Glyma.15G169100.Wm82.a4.v1*) showed unchanged expression after supplementation with nSeB. In the upregulated genes, only one *TCH4* gene (*Glyma.17G065200.Wm82.a4.v1*) showed unchanged expression after supplementation with nSeB, while after the supplementation with nSeA, three genes (*Glyma.13G095200.Wm82.a4.v1*, *Glyma.13G095000.Wm82.a4.v1* and *Glyma.17G065200.Wm82.a4.v1*) were downregulated, and one gene (*Glyma.13G094900.Wm82.a4.v1*) remained unchanged in its expression level (Fig. 5F). In the jasmonic acid signaling pathway, none of the gene showed variation after the supplementation with nSeA and nSeB (Fig. 5G), while in the salicylic acid signaling pathway, one gene (*Glyma.02G283300.Wm82.a4.v1*) in the *nonexpressor of PR1* genes (NPR1), two genes (*Glyma.14G167000.Wm82.a4.v1*, *Glyma.19G130200.Wm82.a4.v1*) in TGACG motif-binding proteins (TGA), and two genes (*Glyma.13G252400.Wm82.a4.v1*, *Glyma.15G062800.Wm82.a4.v1*) in *pathogenesis-related class 1* (PR-1) were upregulated during AsV stress, while their expression level remained unchanged after the supplementation with nSeA. Among these genes, only one *PR-1* gene (*Glyma.15G062800.Wm82.a4.v1*) was downregulated after the supplementation with nSeA, while its expression level remained unchanged after the supplementation with nSeB (Fig. 5H). The above results suggested that supplementation of SeNPs-A regulated plant hormones signaling pathways better than the nSeB and may be involved in modifying the toxicity of AsV stress.

### nSe modulates endogenous phytohormones in root tissues under as toxicity

**Fig. 6:**
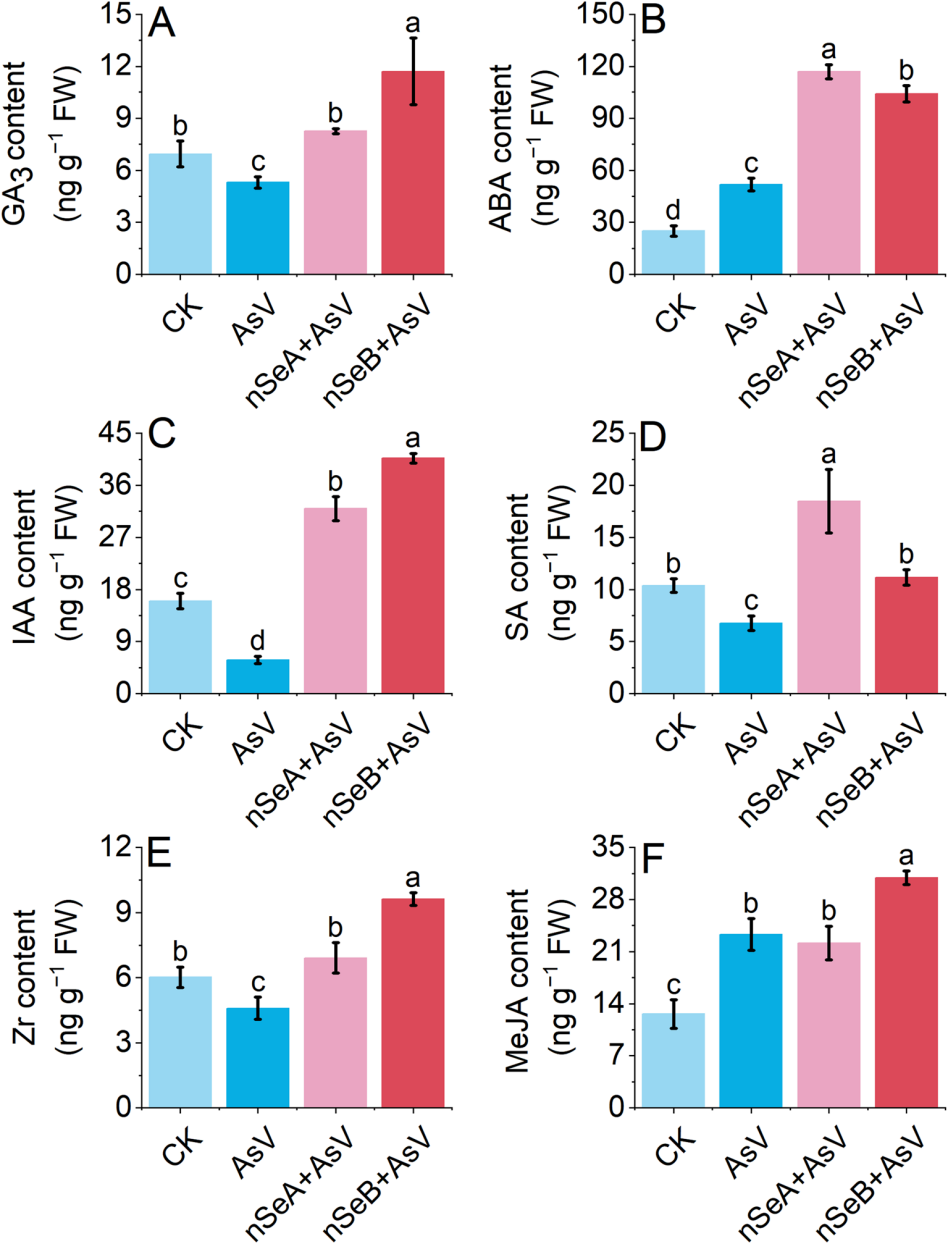
Changes in the concentration of **(A)** gibberellic acid (GA_3_), **(B)** abscisic acid (ABA), **(C)** indole acetic acid (IAA), **(D)** salicylic acid (SA), **(E)** zeatin riboside (ZR), and **(F)** methyl jasmonate (MeJA) in the roots of soybean subjected to AsV and nSe + AsV for 10 days. Data are depicted as mean ± SD for three independent biological replications per treatment. Different small letters above the bars indicate significant differences (*p* < 0.05) using the Tukey HSD test. CK (control), AsV (arsenate stress), nSeA (10 µmol L^− 1^ SeNPs + AsV), nSeB (25 µmol L^− 1^ SeNPs + AsV)

The addition of nSe positively influenced the GA_3_ content in soybean roots subjected to AsV toxicity. For example, AsV stress markedly diminished GA_3_ levels by 28.45% relative to the CK group, whereas nSeB supplementation significantly elevated GA_3_ levels compared to other treatments (Fig. 6A). The GA_3_ content in the presence of nSeA + AsV and nSeB + AsV was 66.24% and 135.57% higher, respectively, than with AsV alone. The ABA content was significantly altered across treatments. AsV stress resulted in a slight but significant increase in ABA levels in soybean root tissue compared to the CK. The introduction of nSe into AsV-stressed plants further increased ABA levels, with nSeA treatment exhibiting a higher ABA content than nSeB (Fig. 6B).

Similarly, relative to the CK treatment, soybean roots treated with AsV significantly reduced the IAA content, whereas the application of nSe dose-dependently increased the level of IAA content. A 63.58% decrease in IAA content was observed under AsV toxicity, while the addition of nSeA and nSeB into AsV-stressed plants increased the IAA level by 99.96% and 154.37%, respectively, compared to the CK plant (Fig. 6C). Also, AsV treatment markedly reduced the SA levels in soybean roots. The supplementation treatment of nSeA significantly improved the SA levels in the AsV-treated plants compared to the CK (Fig. 6D). For instance, nSeA increased the SA level by 76.20% over the AsV-alone treated plant. However, there was no significant increase in SA content between nSeB treatment and AsV treatment.

In addition to these, the Zr and MeJA contents were also modulated under AsV and nSe treatment (Fig. 6E, F). For example, upon AsV stress, Zr content was reduced by 23.75% while MeJA content was increased by 84.60% over the CK treatment. The addition of nSeB into the AsV-stressed plant enhanced the content of these hormones relative to both CK and AsV-alone treatment. There was no significant increase in Zr content between CK and nSeA and in MeJA content between AsV-alone and nSeA treatment.

### Supplementation of nSe switches phenylalanine metabolism and salicylic acid pathway to mitigate AsV toxicity in soybean roots

To further examine the possible role of nSeA in mitigating the AsV stress, pathway analysis showed that taurine and hypotaurine metabolism, phenylalanine metabolism, and sulfur metabolism were only enriched in the CK vs. AsV, CK vs. nSeA, and CK vs. nSeB treatments, respectively (Supplementary Table 3). In the CK vs. nSeB treatment, the enriched sulfur metabolism pathway genes were further studied, and it was found that two genes (*Glyma.05G078300.Wm82.a4.v1* and *Glyma.19G071800.Wm82.a4.v1*) remained unchanged after AsV stress but were significantly downregulated after the supplementation with nSeA and nSeB. In the further stages of sulfur metabolism pathway, only one gene (*Glyma.13G051000.Wm82.a4.v1*) was enriched after the supplementation with nSeA and nSeB. The sulfur metabolism pathway connects to the taurine and hypotaurine metabolism pathway, where five genes (*Glyma.09G258000.Wm82.a4.v1*, *Glyma.09G168900.Wm82.a4.v1*, *Glyma.05G136100.Wm82.a4.v1*, *Glyma.08G091400.Wm82.a4.v1* and *Glyma.08G091500.Wm82.a4.v1*) showed mild variation after the nSeA and nSeB supplementation compared to AsV stress. After several steps, the taurine and hypotaurine metabolism pathway connects to the alpha-linolenic acid pathways, where none of the gene showed variation after the supplementation with nSeA and nSeB conditions.

**Fig. 7:**
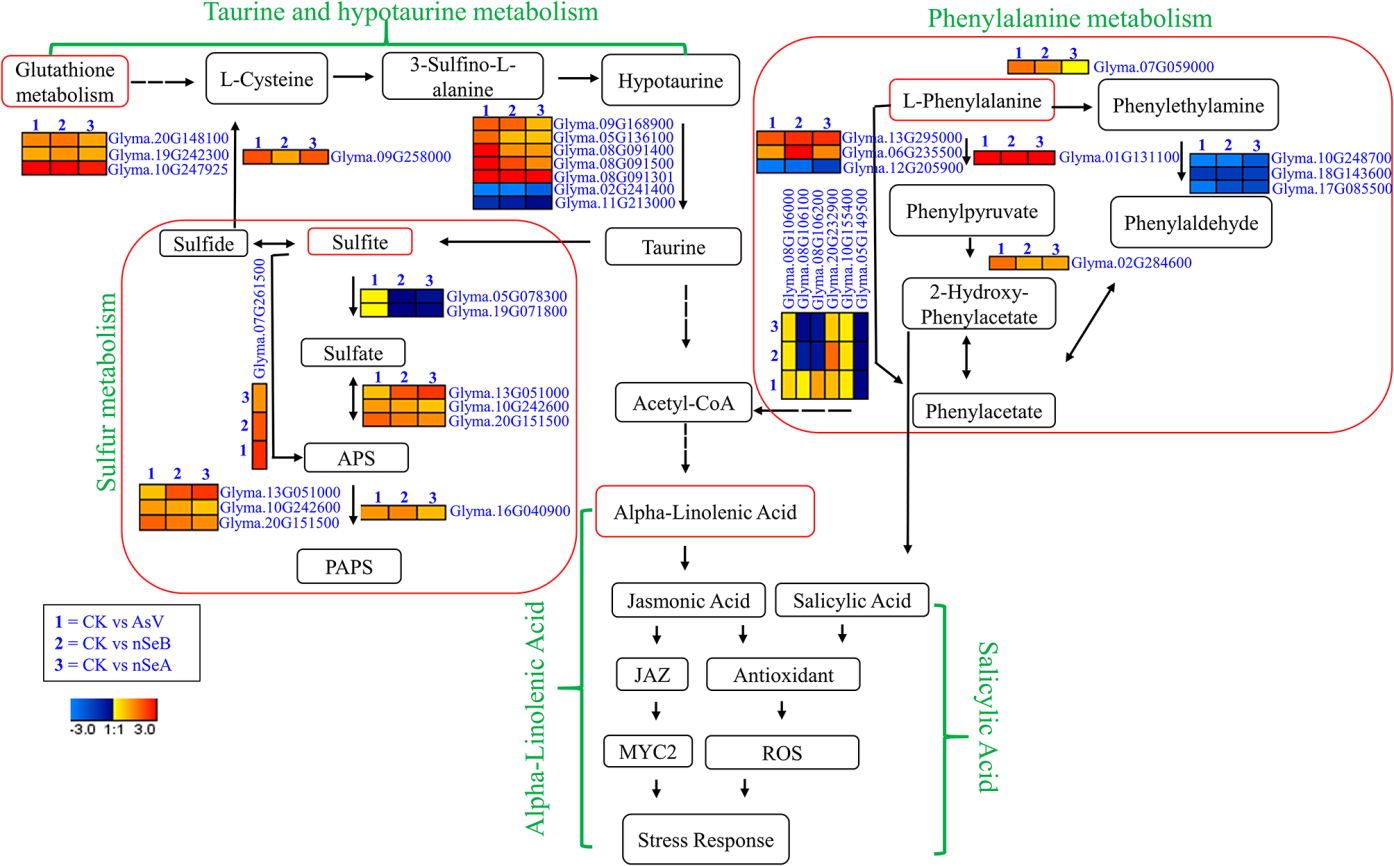
Transcriptional responses of five key metabolic pathways (taurine and hypotaurine metabolism, phenylalanine metabolism, sulfur metabolism, alpha-linolenic acid and salicylic acid) in soybean roots subjected to AsV and nSe treatment. The heatmaps presented depict the log2 fold change values of differentially expressed genes (DEGs), facilitating the visualization of transcriptional alterations across various treatment groups: CK (untreated control), AsV (arsenate stress alone), nSeA (10 µmol L^− 1^ SeNPs plus AsV), and nSeB (25 µmol L^− 1^ SeNPs plus AsV). Reactive oxygen species (ROS), Jasmonate-zim domain (JAZ), MYELOCYTOMATOSIS 2 (MYC2)

In the CK vs. nSeA KEGG enrichment analysis, the phenylalanine metabolism pathway was enriched, which was not enriched in the top 20 pathways of CK vs. AsV and CK vs. nSeB treatments. In the phenylalanine metabolism pathway, five genes were found showing variation after the supplementation with nSeA and nSeB. Among these five genes, two genes (*Glyma.08G106100.Wm82.a4.v1* and *Glyma.08G106200.Wm82.a4.v1*) were downregulated in both nSeA and nSeB supplementation, while two genes (*Glyma.06G235500.Wm82.a4.v1* and *Glyma.20G232900.Wm82.a4.v1*) were upregulated in nSeB supplementation, and only one gene (*Glyma.07G059000.Wm82.a4.v1*) remained unchanged after the supplementation with nSeA. After several steps, the phenylalanine metabolism pathway connects to the salicylic acid pathway, which disrupts the antioxidants and ROS, ultimately affecting the soybean roots. However, supplementation with nSeA increased the antioxidants, which reduced the effect of ROS on soybean roots and, eventually, decreased the stress effect on soybean roots (Fig. 7).

### Analysis of DEGs involved in ROS scavenging under AsV stress

**Fig. 8:**
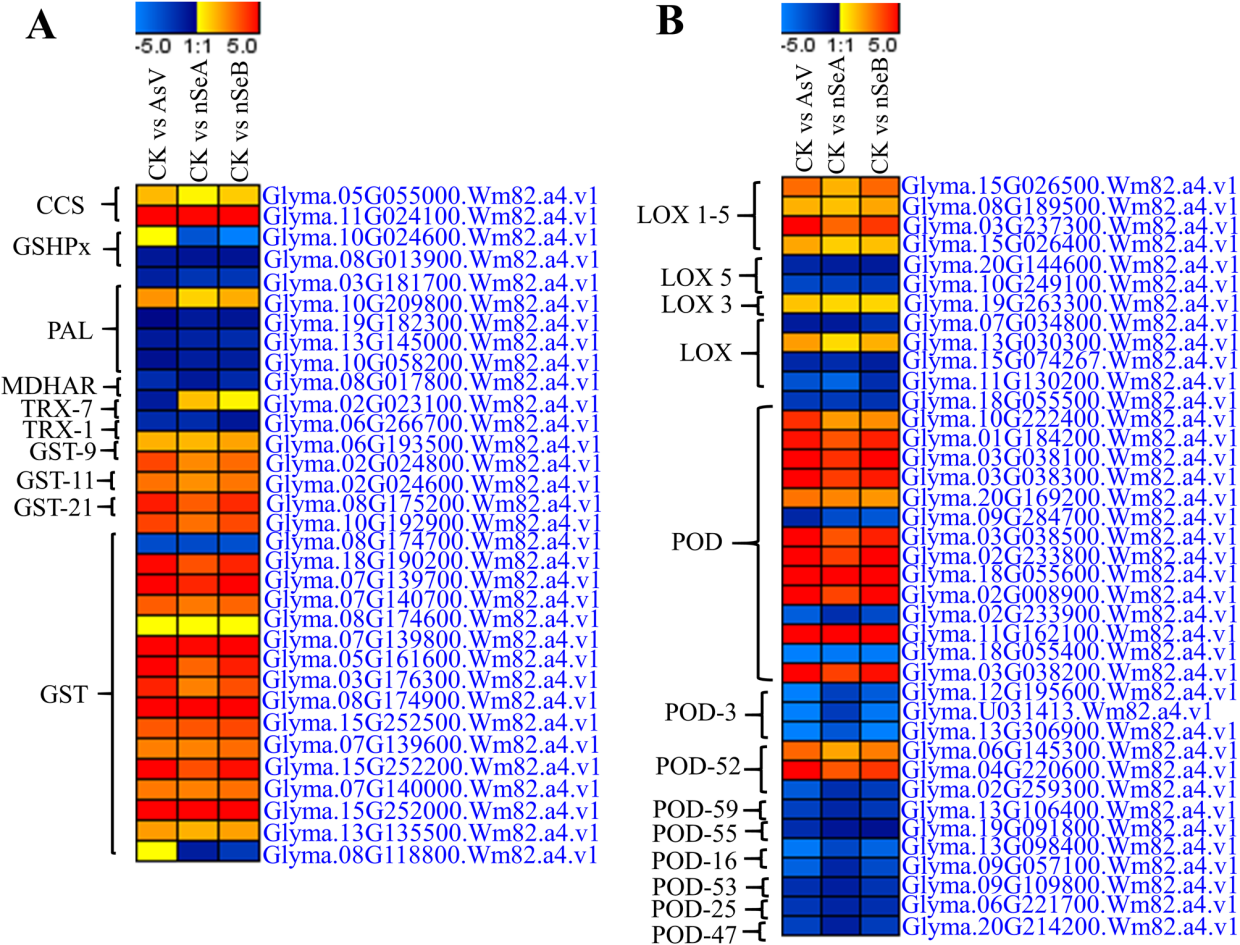
Heat maps of the DEGs related to antioxidant and stress related enzymes under AsV and nSe treatment at different concentration in soybean roots. **(A)** Represent the heatmaps of the expression pattern of DEGs encoding *Superoxide Dismutase* (*CCS*), *Glutathione Peroxidase* (*GSHPx*), *Phenylalanine ammonia-lyase* (*PAL*), *MONODEHYDROASCORBATE REDUCTASE* (*MDHAR*), *THIOREDOXIN* (*TRX-7*), *TRX-1*, *GLUTATHIONE S-TRANSFERASE* (*GST-9*), *GST-11*, *GST-21* and *GST*. **(B)** represent the heatmaps of the expression pattern of DEGs encoding *LIPOXYGENASE* (*LOX 1–5*), *LOX-5*, *LOX-3*, *LOX*, *Peroxidase* (*POD*), *POD-3*, *POD-52*, *POD-55*, *POD-16*, *POD-53*, *POD-25* and *POD-47*. Heatmaps were generated from log_2_ fold change values of DEGs. The various treatment groups include CK (control), AsV (arsenate stress), nSeA (10 µmol L^− 1^ SeNPs + AsV), nSeB (25 µmol L^− 1^ SeNPs + AsV)

To examine the role of nSe in reducing the toxicity created by AsV stress, we collected the expression values of various stress responsive genes from our RNA-seq data (Supplementary Table 4). Among the stress responsive genes, the *Superoxide Dismutase* (CCS) gene (*Glyma.05G055000.Wm82.a4.v1*) was upregulated during AsV stress treatment and remained unchanged after the supplementation with nSeA. The expression data for three *Glutathione Peroxidase* (*GSHPx*) genes were studied, and it was found that only one gene (*Glyma.10G024600.Wm82.a4.v1*) showed variation among the three treatments. The *GSHPx* gene was downregulated after the nSeA and nSeB supplementation, compared to AsV treatment, in which the gene remained unchanged. Among the five *Phenylalanine ammonia-lyase* (*PAL*) genes, only one gene (*Glyma.10G209800.Wm82.a4.v1*) remained unchanged after the supplementation with nSeA, compared to CK treatment, while all the other four genes were downregulated in all treatments. The *THIOREDOXIN* (*TRX1*) gene (*Glyma.02G023100.Wm82.a4.v1*) was downregulated during AsV stress condition and showed clear difference after the supplementation with nSeA and nSeB. Among the seventeen *GLUTATHIONE S-TRANSFERASE* (*GST*) genes, only one gene (*Glyma.08G118800.Wm82.a4.v1*) was downregulated after the supplementation with nSeA and nSeB, while it remained unchanged during the AsV stress condition (Fig. 8A). The other ROS scavenging genes, i.e. *MONODEHYDROASCORBATE REDUCTASE* (*MDHAR*), *TRX-1*, *GST-9*, *GST-11*, *GST-21*, *Peroxidase* (*POD*), *POD-3*, *POD-52*, *POD-59*, *POD-55*, *POD-16*, *POD-53*, *POD-25* and *POD-47*, did not show significant variation after the supplementation with nSeA and nSeB (Fig. 8A, B). To further confirm that the JA signaling pathway does not have a significant role in mitigation of AsV stress after the supplementation with nSe, we also checked the expression of *LIPOXYGENASE* (*LOX*), *LOX1-5*, *LOX-5*, and *LOX-3* genes and found that there was no significant variation found among all treatments (Fig. 8B).

## Discussion

Soybean is an important oilseed as well as nutritionally rich crop, and it is frequently cultivated on marginal as well as on As contaminated land [27, 34], also in the southern provinces of China [26]. To avoid this problem and to solve the issue for facilitating the soybean breeders, it’s necessary to understand the molecular mechanisms associated with As tolerance. In the light of current situation, this study conducted a transcriptomic analysis of soybean roots after being subjected to AsV stress and mitigated with nSe supplementation.

### Modulation of phenylalanine metabolism and salicylic acid pathway to mitigate AsV toxicity in soybean roots

Phenylalanine is an essential amino acid that plays a vital role as a precursor to many molecules important in defense mechanism against biotic and abiotic stresses [35]. It also alleviates the harmful effect of various stresses, such as salinity among others [36]. In our results, KEGG enrichment analysis of CK vs. nSeA showed that the phenylalanine metabolism pathway was enriched, whereas it was not among the top 20 pathways of CK vs. AsV and CK vs. nSeB treatments. In further analysis, we identified 5 genes showing variation after the supplementation with nSeA. Among these 5 genes, 3 genes (*Glyma.08G106100.Wm82.a4.v1*, *Glyma.08G106200.Wm82.a4.v1* and *Glyma.20G232900.Wm82.a4.v1*) are amidase gene that function in plant growth and stress responses [37]. Pérez-Alonso et al. [32] studied the physiological role of *amidase-1* gene in the growth of *Arabidopsis* plants and its adaption to the abiotic stress conditions. They further describe that *amidase-1* have a role in cellular auxin homeostasis by catalyzing the conversion of indole-acetamide into the major plant auxin indole-3-acetic acid. Thus, any effect on the *amidase-1* gene will increase the susceptibility chances of plants to abiotic stresses. In our transcriptomic results, after the supplementation with nSe, the expression of the amidase gene was reduced compared to the AsV stress condition (Fig. 7). *Tyrosine aminotransferase* (TAT) and *Tyrosine decarboxylase* (TDC) were also upregulated and downregulated, respectively, in the phenylalanine metabolism pathway after the supplementation with nSe compared to the AsV stress condition.

Salicylic acid (SA) is an important signaling molecule and plays a crucial role in resistance against biotic and abiotic stress in plants [38]. According to the study of Ding and Ding [39], SA biosynthesis involves two main metabolic pathways: (1) isochorismate pathway and (2) phenylalanine ammonia-lyase pathway. During arsenate stress, the H_2_O_2_ level increases, which ultimately raised the level of ROS [40]. In contrast, SA enhances the antioxidant enzymes, which reduces the ROS level induced by As stress [18]. Sharma et al. [41], also mention that SA treatment boosts the synthesis of key antioxidant enzymes, which reduces the cellular ROS generation and lipid peroxidation in As-stressed plants. Further studies have shown that exogenous application of SA on many plants, including *Artemisia annua* [17], *Glycine max* [19] and *Oryza sativa* [18], promotes plant growth under As-stressed condition [42]. To find the reason we analyzed the endogenous hormones and found that AsV stress reduces the level of SA while after the nSe supplementation, the level of SA increased which ultimately recover the AsV stress (Fig. 6).

Furthermore, the As stress accumulates the ROS which causes oxidative stress and affecting the chlorophyll production as well as photosynthetic activity and leading to the plant growth reduction [21, 43]. In our transcriptomic data, we found that SOD (CCS) gene was up-regulated during the AsV stress while nSeA supplementation normalize its expression and reduce the stress condition, while the GST gene (*Glyma.08G118800.Wm82.a4.v1*) was downregulated after the supplementation with nSeA and nSeB, while it remained unchanged during the AsV stress condition (Fig. 8). SOD is involved in the ROS scavenging enzyme [17] and GSTs have a vital role in detoxifying hydroperoxides and xenobiotics and protect cells from lipid peroxidation [44]. SOD catalyzes the conversion of O^·−2^ to H_2_O_2_ and then converted to O_2_ and water molecules by the action of CAT [20]. Furthermore, in our results, Thioredoxin (*TRX1*) gene (*Glyma.02G023100.Wm82.a4.v1*) was downregulated during AsV stress condition and showed clear difference after the supplementation with nSeA and nSeB (Fig. 7). Ouyang et al. [45] and Zhang et al. [46] also studied a vital role of TRX in modifying hormone signaling, in synthesis of DNA and transcription factors to protect the cells from toxicants. Collectively, these enzymes play a crucial role in the plant defense system, working in coordination to protect cells from AsV stress.

### Hormonal interplay in the presence of nSe involves in AsV tolerance

Biotic and abiotic stresses have a major effect on plant health and to adapt against these stresses, plants require sophisticated sensing, signaling, and response mechanisms. Plant hormones signals are involved in various aspects of development, growth, and environmental stress responses [26, 47, 48]. Disruption of hormonal balance can affect the plant growth. Vezza et al. [27] and Samanta et al. [49] found that As stress disrupts the hormonal balance, which reduces the plant growth. Moreover, the use of SA improves the growth of plant under As stress condition [40]. In various crops such as pepper [50], rosemary [51], and maize [15], it has also been reported that melatonin and SA perform protective functions during As stress condition. In our current transcriptomic results, the salicylic acid signaling pathway showed that one gene (*Glyma.02G283300.Wm82.a4.v1*) from the *NPR1* genes, two genes (*Glyma.14G167000.Wm82.a4.v1*,* Glyma.19G130200.Wm82.a4.v1*) from TGA, and two genes (*Glyma.13G252400.Wm82.a4.v1*,* Glyma.15G062800.Wm82.a4.v1*) from PR-1 were upregulated during AsV stress, while their expression level remained unchanged after the supplementation with nSeA (Fig. 5H).

Except for the SA pathway genes, other hormonal pathway genes, such as those in the auxin signaling pathway, showed that AUX1 genes were downregulated in CK vs. AsV, but their expression were normalized in CK vs. nSeA treatment (Fig. 5A). Likewise, AUX/IAA showed unchanged expression after supplementation with nSeA compared to AsV stress. Further, genes such as SAUR exhibited up- and down-regulation during AsV stress and showed unchanged expression after supplementation with nSeA. The role of auxin during As stress condition in *Arabidopsis* was studied by Krishnamurthy and Rathinasabapathi [52], who found that auxin mutants were sensitive to arsenate stress compared to wild-type plants. In rice, the distribution of auxin was altered by As stress in adventitious as well as lateral roots, affecting the rice plants [53]. Marzi et al. [54] also explained the detail process of auxin response during stress conditions. In the cytokinine, gibberellin, and abscisic acid signaling pathways, the *CRE1*, *GID1*, and *SnRK2s* genes showed unchanged expression after supplementation with nSeA during As stress (Fig. 5B-D). In the ethylene signaling pathway, the *EIN3* and *ERF1/2* genes were upregulated under AsV stress, while both genes showed unchanged expression after supplementation with nSeA (Fig. 5E). The interaction of auxin with other hormones like abscisic acid and ethylene under As stress condition was also studied by others researcher [52]. Ethylene affects the growth of roots by controlling the biosynthesis of auxin [55]. Moreover, it can also control the transport of auxin, performing a negative role in lateral root formation [56] while having a positive role in adventitious root formation [57]. In poplar, Popko et al. [58] discussed the crosstalk between auxin and abscisic acid during stress conditions.

Based on the above study, it is hypothesized that during arsenate stress, the H_2_O_2_ level increases, ultimately leading to an increase in ROS level. However, after the supplementation of nSeA in AsV stress plants, the toxicity of AsV stress is mitigated by controlling the hormonal signaling pathway as well as recovering the genes expression values in phenylalanine metabolism and SA biosynthesis pathways, which increase the antioxidants and reduce the effect of ROS on soybean roots. This eventually decreases the stress effect on the soybean roots. However, further studies are needed to explore the specific role of hormonal interplay in soybean roots under the supplementation of SeNPs during AsV stress conditions.

## Conclusion

In conclusion, our study demonstrates the potential of nSe to mitigate As toxicity in soybean roots by modulating key metabolic and signaling pathways, particularly the phenylalanine and salicylic acid pathways. Our findings highlight that nSe enhance antioxidant activity and reduce reactive oxygen species (ROS) damage, providing a protective mechanism against As stress in soybean seedlings. The results offer valuable insights into the molecular basis of metalloid tolerance in plants and suggest that nSe could serve as a promising tool for improving crop resilience in As-contaminated soils. While this research broadens our understanding of the interaction between Se and As in plants, further investigation is required to fully unravel the role of hormonal signaling and its interaction with nSe during As stress. Such insights will be critical for advancing agricultural practices in regions where soil contamination poses a significant challenge.

## Electronic supplementary material

Below is the link to the electronic supplementary material.

Supplementary Material 1

## Data Availability

The original contributions presented in the study are deposited in the Sequence Read Archive (SRA) repository, accession number PRJNA1247022. Further inquiries can be directed to the corresponding authors.
